# Coronary pressure and flow relationships in humans: phasic analysis of normal and pathological vessels and the implications for stenosis assessment: a report from the Iberian–Dutch–English (IDEAL) collaborators

**DOI:** 10.1093/eurheartj/ehv626

**Published:** 2015-11-26

**Authors:** Sukhjinder S. Nijjer, Guus A. de Waard, Sayan Sen, Tim P. van de Hoef, Ricardo Petraco, Mauro Echavarría-Pinto, Martijn A. van Lavieren, Martijn Meuwissen, Ibrahim Danad, Paul Knaapen, Javier Escaned, Jan J. Piek, Justin E. Davies, Niels van Royen

**Affiliations:** 1 Imperial College London , London , UK; 2 VU University Medical Centre , Amsterdam , The Netherlands; 3 Academic Medical Centre , Amsterdam , The Netherlands; 4 Cardiovascular Institute , Hospital Clínico San Carlos , Madrid , Spain; 5 Amphia Hospital , Breda , The Netherlands

**Keywords:** Autoregulation, Microvascular resistance, Physiological lesion assessment, Stenosis

## Abstract

**Background:**

Our understanding of human coronary physiological behaviour is derived from animal models. We sought to describe physiological behaviour across a large collection of invasive pressure and flow velocity measurements, to provide a better understanding of the relationships between these physiological parameters and to evaluate the rationale for resting stenosis assessment.

**Methods and results:**

Five hundred and sixty-seven simultaneous intracoronary pressure and flow velocity assessments from 301 patients were analysed for coronary flow velocity, trans-stenotic pressure gradient (TG), and microvascular resistance (MVR). Measurements were made during baseline and hyperaemic conditions. The whole cardiac cycle and the diastolic wave-free period were assessed. Stenoses were assessed according to fractional flow reserve (FFR) and quantitative coronary angiography DS%. With progressive worsening of stenoses, from unobstructed angiographic normal vessels to those with FFR ≤ 0.50, hyperaemic flow falls significantly from 45 to 19 cm/s, *P*_trend_ < 0.001 in a curvilinear pattern. Resting flow was unaffected by stenosis severity and was consistent across all strata of stenosis ( *P*_trend_ > 0.05 for all). Trans-stenotic pressure gradient rose with stenosis severity for both rest and hyperaemic measures ( *P*_trend_ < 0.001 for both). Microvascular resistance declines with stenosis severity under resting conditions ( *P*_trend_ < 0.001), but was unchanged at hyperaemia (2.3 ± 1.1 mmHg/cm/s; *P*_trend_ = 0.19).

**Conclusions:**

With progressive stenosis severity, TG rises. However, while hyperaemic flow falls significantly, resting coronary flow is maintained by compensatory reduction of MVR, demonstrating coronary auto-regulation. These data support the translation of coronary physiological concepts derived from animals to patients with coronary artery disease and furthermore, suggest that resting pressure indices can be used to detect the haemodynamic significance of coronary artery stenoses.

**See page 2081 for the editorial comment on this article (doi:10.1093/eurheartj/ehv688)**

## Introduction

 The physiological behaviour of human coronary stenoses has been inferred from animal experiments that studied changes of flow velocity and pressure in the presence of artificially created stenoses. ^1–3^ These experiments determined that stenoses created by external constriction or ligation had a non-linear relationship between the degree of coronary narrowing and trans-stenotic flow velocity and pressure gradient. ^3^ Early attempts to replicate this across patients with coronary artery disease were unsuccessful, presumably due to the effect of atherosclerosis and cardiovascular risk factors on other domains of the coronary circulation. ^4 , 5^ Nevertheless, animal models continue to be used to describe human physiology. 

 Since plotting pressure–flow relationships can be challenging in clinical practice, indices have been formulated to describe the importance of stenoses. ^6 , 7^ Those involving both pressure and flow velocity measurement have predominantly been used in a research setting. ^7–10^ Pressure-only measurements, being easier to perform, have gained more common clinical application. Fractional flow reserve (FFR), a pressure-only hyperaemic measure is treated as a simplified surrogate for flow based upon assessments in animals. It has compelling outcome data and is widely advocated to guide coronary assessment. ^11^ Another newer pressure-only index, instantaneous wave-free ratio (iFR) ^12 , 13^ is measured under resting conditions, obviating the need for hyperaemic vasodilators such as adenosine. While the rationale for the hyperaemic physiological assessment of coronary artery stenosis has been extensively validated, resting stenosis assessment has been less extensively explored. 

The aim of this study is to investigate the coronary pressure–flow relationship in patients with and without angiographic evidence of obstructive atherosclerosis under resting and hyperaemic conditions. The IDEAL dataset is used to analyse 567 human coronary artery intracoronary pressure and flow velocity recordings to revisit pressure–flow relationships in a large clinical cohort of patients with stable coronary artery disease, representative of the clinical ‘real world’.

## Methods

### Study population

 This study incorporates prospectively collected data from a total of 567 combined pressure and Doppler flow velocity measurements in 301 patients at the Amsterdam Medical Center Amsterdam, The Netherlands ( *n* = 161), Imperial College London, UK ( *n* = 160), Hospital Clinico San Carlos, Madrid, Spain ( *n* = 21), and VU University Medical Center, Amsterdam, The Netherlands ( *n* = 225). All patients recruited were scheduled for elective coronary angiography with physiological stenosis assessment by FFR and gave written informed consent for acquisition of additional physiological data for study purposes. 

 While acquisition methodology of physiological data was similar for all participating centres, the study protocol was different for each centre. Individual centre recruitment criteria are shown in Supplementary material online . Composite exclusion criteria were severe valvular heart disease, weight >200 kg (determined by the catheter laboratory table capacity), previous coronary artery bypass surgery, vessels with angiographically identifiable myocardial bridging or collateral arteries and vessels with a previous myocardial infarction. Patients with an acute myocardial infarction within 48 h were not included. 

### Coronary catheterization

 Coronary angiography and pressure–flow assessments of coronary stenoses were performed using conventional approaches. ^14^ Intracoronary nitrates (200–300 μg) were administered in all cases. Contemporary combined pressure and Doppler flow velocity wires (ComboWire XT, Volcano Corporation, San Diego, CA, USA) were used and the distal pressure sensor was equalized with the aortic guiding pressure at the coronary ostium before distal passing of the wire. Measurements were made distal to the stenosis at least three vessel diameters from the stenosis. Adenosine was administered by intravenous infusion in 234 measurements (140 μg/kg/min) and by intracoronary bolus injection in 333 measurements (60–150 μg). 

Doppler signals were optimized carefully to ensure adequate tracking profiles were observed. Electrocardiogram (ECG), pressures, and flow velocity signals were directly extracted from the device console (ComboMap, Volcano Corporation, San Diego, CA, USA). At the end of each recording, the pressure sensor was returned to the catheter tip to assess pressure drift. If pressure drift was identified (>2 mmHg) measurements were repeated or corrected for upon analysis. Data were analysed off-line, using a custom software package designed with MATLAB (Mathworks, Inc, Natick, MA, USA). A total of 653 cases were originally acquired, but 86 vessels (13.2%) were excluded because of poor Doppler flow velocity or uncontrollable pressure drift leaving 567 vessels for final analysis. Resting indices were calculated at a time of stability, without any preceding injection of contrast or saline. Hyperaemic indices were calculated during stable hyperaemia, excluding ectopy and conduction delay.

### Stenosed and reference vessels

Five hundred and sixty-seven coronary assessments were made. Three hundred and sixty-six vessels had an angiographically visible stenosis. Two hundred and one vessels had no angiographic obstruction as determined by the physician performing the procedure and confirmed by two observers (S.N. and G.d.W.).

### Stenosis stratification

Both FFR and diameter stenosis assessed by quantitative angiography (QCA) were used to stratify stenosis severity. Myocardial FFR measurements were performed, using the ratio of distal coronary pressure to proximal pressure during stable hyperaemia. Quantitative angiography parameters (diameter stenosis % (DS%), minimal lumen diameter, minimal lumen area, area stenosis, and lesion length) were calculated for stenoses using dedicated workstations (CAAS II, Pie Medical, Maastricht, The Netherlands or McKesson, San Francisco, USA), which performed automated contour analysis with manual correction limited to situations causing artefacts or very tight stenoses.

### Calculation of hemodynamic parameters

**Figure 1:**
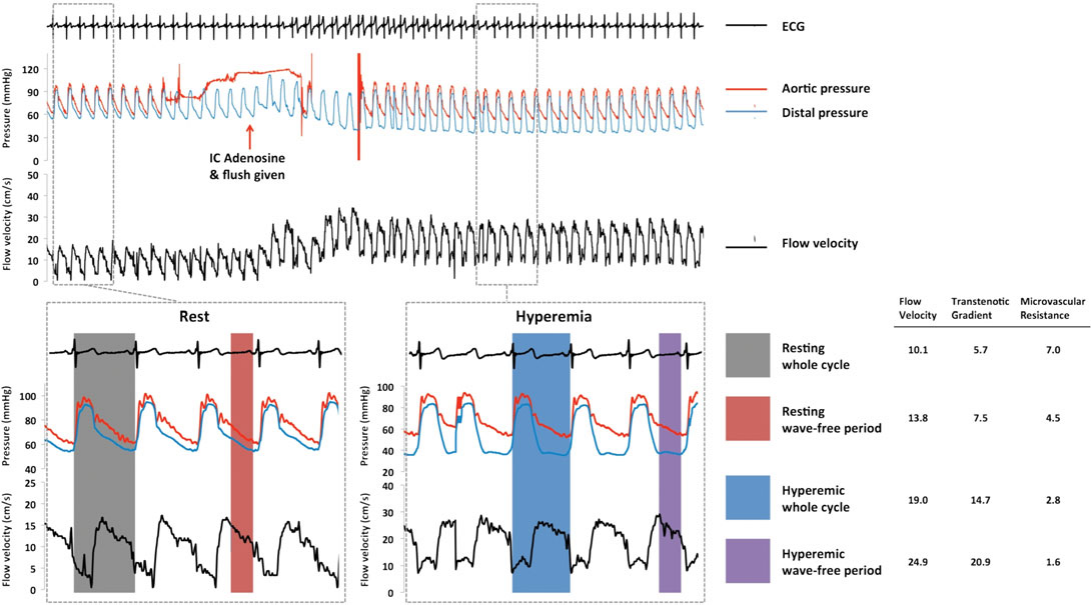
Top panel: example of simultaneous coronary pressure and flow velocity measurement obtained distal to an left anterior descending stenosis of 69% diameter stenosis by quantitative angiography with FFR of 0.79. Bottom panel: the analysed phases during the resting and hyperaemic state are shown.

 Flow velocity was assessed over four periods: first, flow velocity at rest over the entire cardiac cycle and secondly over the specific diastolic wave-free period (during which iFR is calculated), which was detected using the ECG signals. Flow velocity was also assessed during adenosine-mediated hyperaemia over the whole cardiac cycle and the wave-free period. The same two time periods in the cardiac cycle, both at rest and hyperaemia, were used to derive measures of microvascular resistance (MVR) and trans-stenotic pressure gradient (TG). * Figure  1* shows an example of simultaneous pressure and flow velocity measurements, together with the cardiac phases over which the study parameters were calculated. 

Microvascular resistance (mmHg/cm/s) is calculated by dividing distal pressure (Pd, mmHg) by flow velocity (cm/s). When calculated for the whole cardiac cycle, values of pressure and flow velocity were averaged over an entire heart beat; typically measurements were made as an average over five beats. When calculated over the wave-free period, pressure and flow velocity data were constrained to that averaged over the diastolic wave-free period.

 For all measurements, computation of the parameters was performed by a single analyst blinded to the coronary angiograms or patient specific factors, using an automated MATLAB script (MathWorks, Natick, MA, USA) with built-in wave-free algorithm (developed at Imperial College, London and licensed to Volcano Corp, San Diego, CA, USA), as previously described. ^12^

### Statistical analysis

 Categorical data are presented as numbers and percentages, while continuous data are presented as mean ± standard deviation. Regression analysis was performed between quantitative values to determine the coefficient of determination. Curve fitting was achieved by applying 2nd and 3rd order and fractional polynomials. Association between flow velocity (dependent variable) and strata of stenosis severity were assessed by analysis of variance (ANOVA) with correction for repeated measures followed by *post hoc* pairwise methods, including Bonferroni, Sidak, and Scheffe; this was followed by Tukey HSD testing where appropriate. Findings were confirmed using Kruskal–Wallis testing to avoid assumptions of normality. Trends across strata were assessed with regression and a non-parametric extension of the Wilcoxon signed rank test (nptrend) and also with generalized estimating equations; in all analyses, the findings were the same suggesting a robust analysis. The analysis was repeated for TG or MVR as dependent variables. A *P* -value of <0.05 was considered statistically significant. All statistical analyses were performed using Stata 11.2 (StataCorp, TX, USA). 

## Results

### Patient and vessel characteristics

 Five hundred and sixty-seven coronary assessments were derived from 301 patients (age 53.5 ± 22.3 years old, 59% male, * Table  1* ). No patients had hypertrophic cardiomyopathy or gross hypertrophy secondary to hypertension. Characteristics of patients and vessels is shown per participating centre in Supplementary material online, Table S1 . Three hundred and sixty-six (65%) were from vessels with a visible stenosis, with 85 measurements taken post-percutaneous coronary intervention (PCI). Two hundred and one (35%) measurements were from vessels free from angiographic disease, which served as reference vessels. For the 366 vessels with a stenosis, the FFR ranged from 0.28 to 1.07. 

**Table 1:** Demographics and stenosis characteristics

	N or mean	% or standard deviation
Patients	**301**	
Age (years)	60.6	9.6
Male	209	69%
Hypertension	157	52%
Hyperlipidemia	172	57%
Current or ex-smoker	128	43%
Diabetes Mellitus	67	22%
Chronic renal impairment	5	2%
Family history of CAD	129	43%
Previous myocardial infarction	34	11%
Impaired LV function EF < 30%	2	0.7%
Stable angina	290	96%
Unstable angina	11	4%
Vessels	**567**	
Angiographic stenosis	366	65%
Angiographically unobstructed	201	35%
Coronary artery		
Left Anterior Descending	277	49%
Left Circumflex	172	30%
Right Coronary Artery	118	21%
Adenosine administration		
Central intravenous	234	41%
Intracoronary bolus	333	59%
Coronary stenoses		
% Diameter stenosis	46.0	21.3
% Area stenosis	68.9	22.8
Minimal lumen diameter (mm)	1.47	0.75
Minimal lumen area (mm ^2^ )	2.09	2.21
Stenosis length (mm)	17.0	12.5

**Figure 2:**
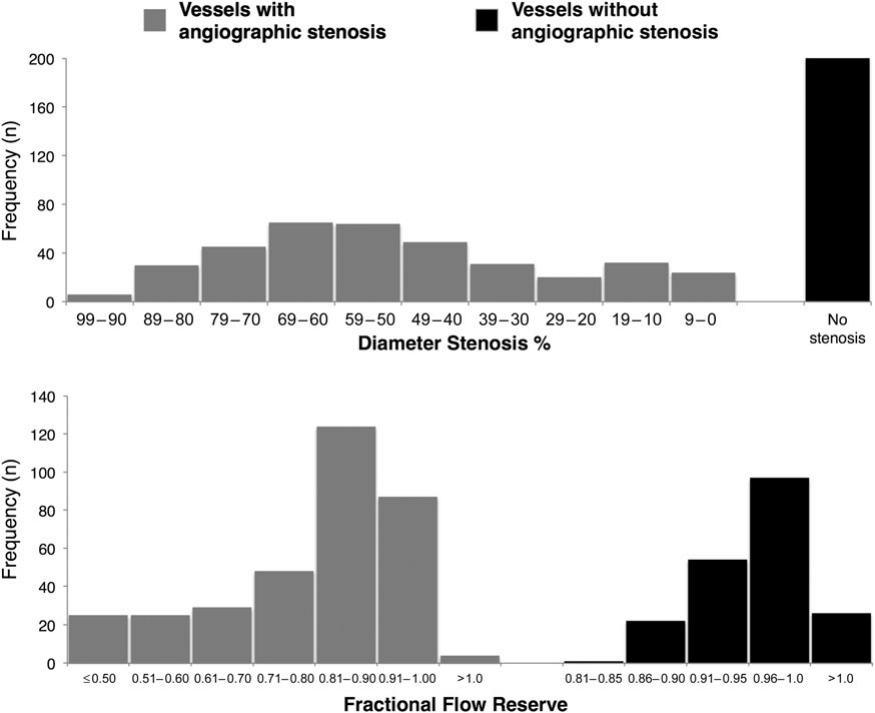
Distribution of the coronary arteries measured stratified according to percentage of diameter stenosis % (upper panel) and fractional flow reserve (lower panel).

**Figure 3:**
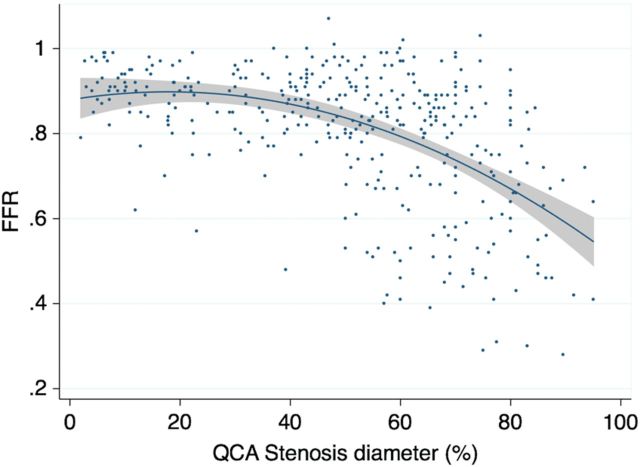
Distribution of percentage of diameter stenosis and fractional flow reserve in stenoses. Despite a significant inverse correlation between percentage of diameter stenosis and fractional flow reserve, a substantial variability between the two parameters is noted ( *R*^2^ = 0.23, *P* < 0.01). The curve is fitted by second-order polynomial.

 The distribution of stenoses was consistent with that typically found in clinical practice with mean FFR 0.81 ± 0.16 and mean QCA diameter stenosis of 48.5 ± 25.2%, indicating that the majority was of intermediate severity ( * Figure  2* ). In the 201 reference vessels, FFR ranged from 0.85 to 1.08 with a mean FFR of 0.96 ± 0.04. Overall, these findings confirmed the absence of obstructive epicardial disease. In 23 (11%) of the reference cases, an FFR of >0.80 and ≤0.90 was documented, suggesting the existence of abnormal epicardial conductance. A value of FFR > 1.0 was noted in 30 vessels despite careful drift assessment. This predominantly occurred in the LCx (18 cases, 60%) and in reference vessels (26 cases, 87%) and was likely due to hydrostatic consequences of the wire being in a distal vessel below the position of the transducer. For the stenosed vessels, the relationship between FFR and anatomical DS% demonstrated significant scatter ( *R*^2^ = 0.23, *P* < 0.01; * Figure  3* ). Similar findings were noted when plotting FFR and anatomical minimal luminal diameter ( *R*^2^ = 0.19, *P* < 0.001; Supplementary material online, Figure S1 ). 

### Whole cycle pressure–flow velocity relationships

**Figure 4:**
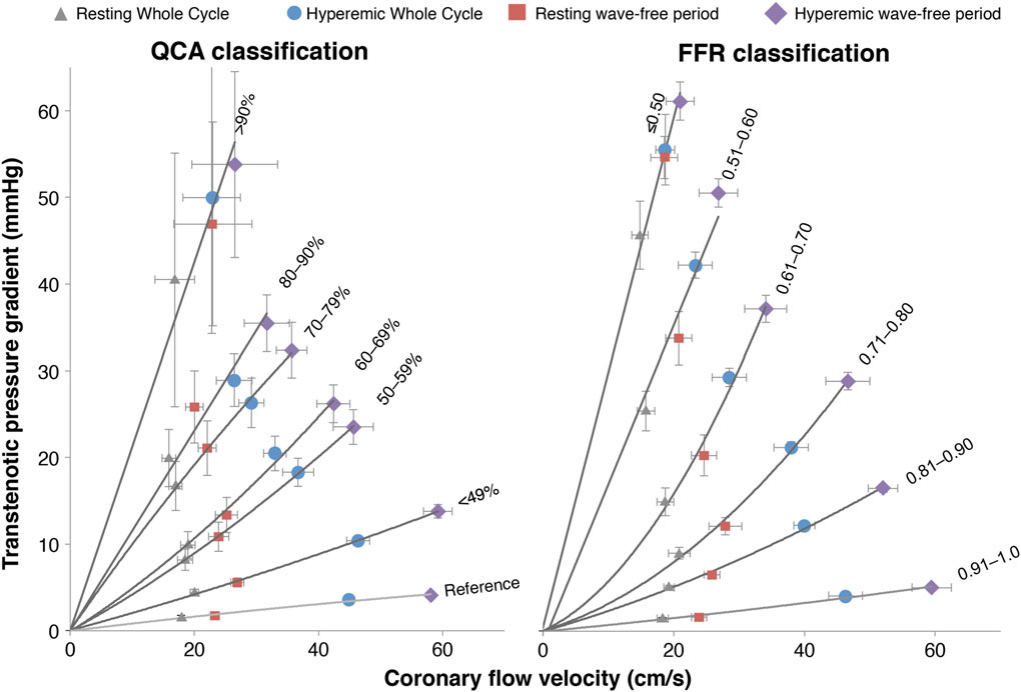
Relationships between trans-stenotic pressure gradient and flow velocity for coronaries grouped by stenosis severity (left panel according to anatomical severity by percentage of diameter stenosis, and right panel according to physiological severity by fractional flow reserve). Relationships are described by trans-stenotic pressure gradient = A* flow + B* flow ^2^ and can be fitted by three points: the zero TG—zero flow crossing, the mean trans-stenotic pressure gradient and flow during whole cycle at rest, and during hyperaemia. Trans-stenotic pressure gradient and flow during the wave-free period closely follow these relationships, both at rest and during hyperaemic conditions. Curves are fitted by second-order polynomials.

 A given stenosis has a unique curvilinear relationship between the flow velocity and the TG across the stenosis. Pressure–flow velocity relationships over the whole cardiac cycle were calculated both during resting and hyperaemic conditions, and averaged for each stratum of stenosis severity. Mean pressure–flow velocity relationships were stratified according to DS% ( * Figure  4* , left panel) and according to FFR classification ( * Figure  4* , right panel). 

### Diastolic pressure and flow velocity relationships

 Pressure–flow velocity relationships were calculated over the wave-free period specifically, both at rest and hyperaemia. For each stratification, these relationships closely fit the pressure–flow velocity curves derived from whole-cycle physiology ( * Figure  4* ). 

 Resting wave-free period flow velocity was significantly higher than whole cycle resting conditions ( *P* < 0.05 for each FFR or QCA stratum), and consistently produced a higher TG, both at rest and during hyperaemia ( *P* < 0.05 for each FFR or QCA stratum). The only exception was TG in reference vessels and FFR > 0.91 under resting conditions, where TG was equivalent for whole cycle and wave-free period (1.5 ± 0.2 vs. 1.6 ± 0.2 mmHg, respectively; *P* = 0.53, and 1.1 ± 0.1 vs. 1.3 ± 0.1 mmHg; *P* = 0.08) as there was no stenosis sufficient to cause diastolic pressure separation. Under hyperaemic conditions, both a consistently higher flow velocity and TG were found during the wave-free period than during whole cycle ( *P* < 0.05 for each FFR or QCA stratum). 

### Influence of stenosis severity on coronary flow velocity

**Figure 5:**
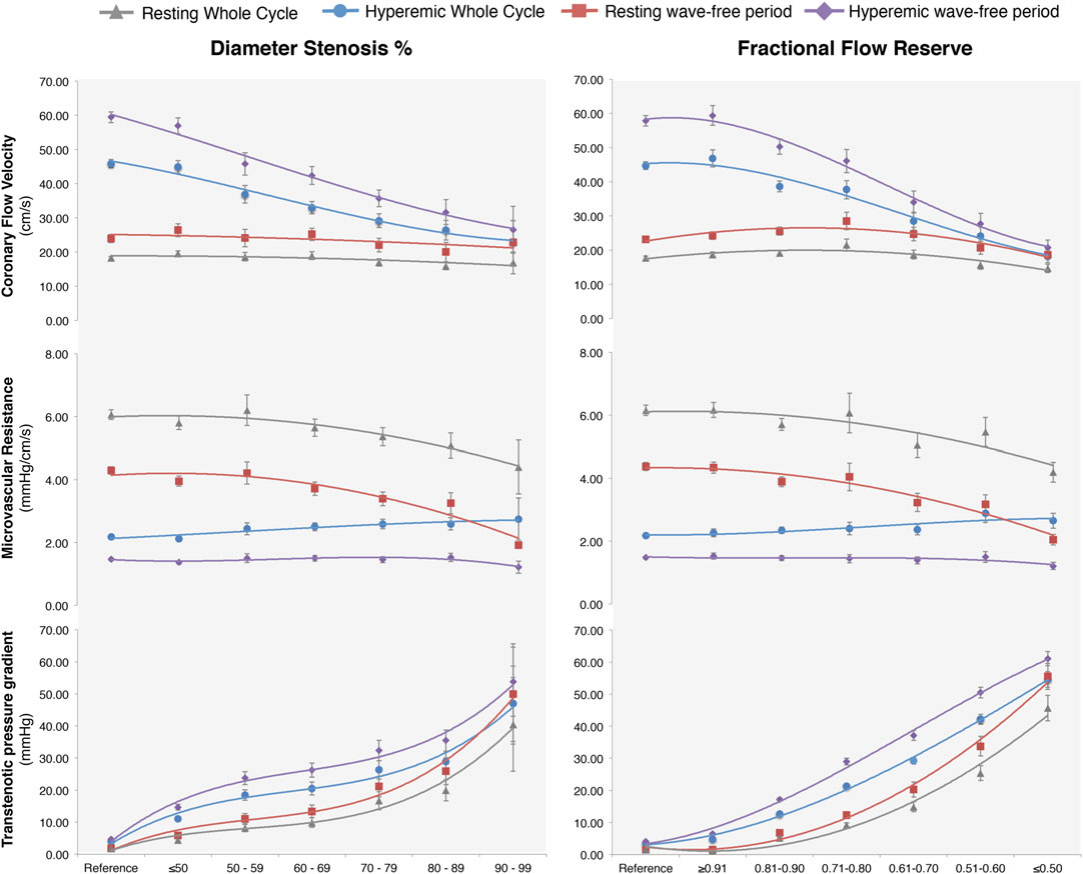
Behaviour of phasic coronary flow velocity, microvascular resistance (MVR) and (TG) according to stenosis severity (left panel diameter stenosis by QCA, and right panel by FFR). Parameters are shown for resting and hyperemic conditions, both during whole cycle and wave-free period only. Curves are fitted by second-, third-order, and fractional polynomials.

 Resting flow velocity stratified according to angiographic and FFR strata is depicted in * Figure  5* , upper panel. Numerical relationships between stenosis severity and the analysed parameters, as well as the physiological indices, are shown in * Tables  2 and 3* . Resting flow velocity has no significant relationship with stenosis severity whether assessed by FFR or anatomical severity ( *P*_trend_ = 0.16). Hyperaemic flow velocity, over the whole cycle and the wave-free period, shows a strong statistical association and trend to decline with incremental stenosis severity ( *P* < 0.001 for all assessments). 

**Table 2:** Flow velocity, TG, MVR and physiological indices according to lesion severity defined by FFR

	FFR ≤ 0.50	SD	FFR 0.51–0.60	SD	FFR 0.61–0.70	SD	FFR 0.71–0.80	SD	FFR 0.81–0.90	SD	FFR >0.91	SD
Flow velocity (cm/s)												
Resting whole cycle	**14.8**	6.3	**15.5**	7.1	**18.6**	6.7	**21.9**	12.1	**18.9**	8.7	**19.1**	8.4
Resting wave-free period	**18.7**	7.4	**20.3**	10.4	**24.6**	10.1	**29.1**	18.3	**25.4**	13.2	**25.1**	11.4
Hyperemic whole cycle	**18.5**	10.3	**22.8**	13.6	**28.3**	13.7	**36.9**	18.9	**38.9**	18.0	**48.2**	23.7
Hyperemic wave-free period	**21.0**	10.8	**25.7**	14.6	**34.1**	16.9	**45.0**	24.6	**50.4**	24.8	**61.4**	28.0
Trans-stenotic gradient (mmHg)												
Resting whole cycle	**45.6**	19.7	**26.0**	11.8	**15.5**	8.6	**9.81**	5.0	**5.19**	3.06	**1.17**	2.32
Resting wave-free period	**55.5**	20.3	**35.1**	15.8	**20.7**	12.3	**13.2**	7.56	**6.88**	4.01	**1.48**	2.43
Hyperemic whole cycle	**54.6**	12.1	**42.6**	7.86	**30.17**	6.14	**22.2**	4.63	**13.0**	4.71	**4.71**	3.19
Hyperemic wave-free period	**61.1**	11.0	**51.2**	8.49	**37.9**	8.40	**30.4**	7.37	**17.6**	5.65	**6.34**	4.25
Microvascular Resistance (mmHg/cm/s)												
Resting whole cycle	**4.20**	1.57	**5.55**	2.40	**5.04**	2.03	**5.61**	4.34	**5.80**	2.40	**6.09**	2.39
Resting wave-free period	**2.04**	0.83	**3.22**	1.62	**3.19**	1.52	**3.76**	3.01	**3.93**	1.82	**4.27**	1.79
Hyperemic whole cycle	**2.65**	1.19	**2.97**	1.55	**2.37**	0.88	**2.36**	1.37	**2.32**	1.10	**2.20**	1.12
Hyperemic wave-free period	**1.22**	0.56	**1.55**	0.90	**1.37**	0.62	**1.41**	0.88	**1.45**	0.81	**1.49**	0.86
Indices												
Pd/Pa	**0.56**	0.15	**0.74**	0.11	**0.84**	0.07	**0.91**	0.05	**0.95**	0.03	**0.99**	0.02
iFR	**0.39**	0.18	**0.60**	0.17	**0.76**	0.12	**0.84**	0.10	**0.92**	0.05	**0.98**	0.03
FFR	**0.42**	0.06	**0.54**	0.03	**0.66**	0.03	**0.75**	0.03	**0.85**	0.03	**0.95**	0.03
HSR (mmHg/cm/s)	**3.92**	2.36	**2.49**	1.30	**1.23**	0.46	**0.78**	0.51	**0.40**	0.21	**0.12**	0.11
BSR (mmHg/cm/s)	**3.83**	2.58	**2.02**	1.25	**0.88**	0.44	**0.55**	0.44	**0.32**	0.23	**0.07**	0.16
CFR	**1.26**	0.44	**1.51**	0.69	**1.61**	0.71	**1.81**	0.54	**2.16**	0.74	**2.62**	0.88
Anatomical parameters												
% Diameter stenosis	**71.3**	12.4	**65.0**	13.3	**60.0**	18.6	**51.8**	18.0	**43.3**	18.7	**33.4**	18.6
% Area stenosis	**90.1**	8.36	**87.8**	9.26	**78.0**	19.0	**77.2**	17.7	**66.7**	22.6	**57.7**	22.6
Minimal lumen diameter (mm)	**0.80**	0.39	**0.93**	0.29	**1.01**	0.42	**1.20**	0.46	**1.53**	0.72	**1.87**	0.79
Minimal lumen area (mm ^2^ )	**0.63**	0.55	**0.76**	0.46	**0.94**	0.83	**1.26**	0.99	**2.23**	2.27	**3.13**	2.57
Stenosis length (mm)	**26.7**	16.1	**32.4**	18.1	**20.9**	18.2	**19.5**	13.3	**15.0**	9.05	**12.9**	8.79

**Table 3:** Flow velocity, TG, MVR and physiological indices according to lesion severity defined by anatomical stenosis severity (% diameter stenosis)

	QCA ≥ 90%	SD	QCA 80–89%	SD	QCA 70–79%	SD	QCA 60–69%	SD	QCA 50–59%	SD	QCA ≤ 49%	SD	Reference	SD
Flow Velocity (cm/s)														
Resting whole cycle	**16.4**	8.02	**17.5**	6.52	**14.9**	7.60	**19.4**	9.63	**17.7**	9.0	**20.0**	8.9	**17.8**	6.9
Resting wave-free period	**23.7**	12.6	**21.4**	7.63	**19.5**	10.9	**25.3**	14.4	**23.1**	12.1	**26.5**	12.8	**23.3**	10.2
Hyperemic whole cycle	**24.7**	17.9	**24.1**	11.0	**23.5**	12.2	**31.1**	14.2	**35.0**	19.0	**45.4**	22.3	**44.9**	16.0
Hyperemic wave-free period	**28.8**	19.2	**27.7**	11.5	**28.8**	16.9	**38.6**	19.5	**44.4**	25.0	**57.8**	27.9	**58.1**	21.6
Trans-stenotic gradient (mmHg)														
Resting whole cycle	**34.7**	26.1	**29.5**	27.2	**22.8**	19.1	**13.4**	16.1	**8.10**	9.36	**4.42**	4.97	**1.53**	2.51
Resting wave-free period	**47.4**	31.9	**35.9**	29.8	**28.8**	22.4	**17.9**	19.3	**10.8**	12.7	**5.64**	6.72	**1.58**	2.82
Hyperemic whole cycle	**41.9**	18.5	**37.5**	23.2	**32.4**	18.2	**24.9**	17.9	**18.0**	12.4	**10.8**	7.72	**3.55**	4.02
Hyperemic wave-free period	**52.1**	18.0	**43.1**	23.5	**39.3**	19.3	**31.3**	19.4	**23.5**	15.2	**14.2**	10.1	**4.04**	5.28
Microvascular Resistance (mmHg/cm/s)														
Resting whole cycle	**4.9**	2.37	**4.22**	1.60	**5.50**	2.29	**5.30**	2.13	**6.27**	3.55	**5.73**	2.46	**6.16**	2.33
Resting wave-free period	**1.93**	0.31	**2.45**	0.97	**3.38**	1.91	**3.41**	1.61	**4.25**	2.51	**3.92**	1.85	**4.38**	1.83
Hyperemic whole cycle	**2.84**	1.91	**2.65**	1.28	**2.69**	0.99	**2.52**	1.10	**2.56**	1.50	**2.14**	1.01	**2.18**	0.80
Hyperemic wave-free period	**1.07**	0.37	**1.44**	0.67	**1.49**	0.71	**1.47**	0.71	**1.58**	1.08	**1.38**	0.75	**1.48**	0.62
Indices														
Pd/Pa	**0.68**	0.21	**0.72**	0.21	**0.77**	0.19	**0.87**	0.15	**0.92**	0.10	**0.96**	0.05	**0.98**	0.03
iFR	**0.51**	0.28	**0.60**	0.27	**0.67**	0.24	**0.80**	0.21	**0.87**	0.15	**0.93**	0.08	**0.98**	0.03
FFR	**0.55**	0.16	**0.60**	0.17	**0.64**	0.18	**0.73**	0.17	**0.80**	0.14	**0.88**	0.09	**0.96**	0.04
HSR (mmHg/cm/s)	**3.33**	3.52	**2.34**	2.60	**1.22**	1.50	**0.78**	1.03	**0.78**	1.03	**0.32**	0.32	**0.09**	0.10
BSR (mmHg/cm/s)	**3.26**	3.32	**2.22**	2.68	**0.91**	1.31	**0.55**	0.79	**0.55**	0.79	**0.25**	0.27	**0.09**	0.17
CFR	**1.37**	0.38	**1.36**	0.36	**1.65**	0.67	**1.67**	0.59	**2.12**	0.85	**2.39**	0.86	**2.64**	0.76

Hyperemic stenosis resistance (HSR) is defined as the ratio between the TG and flow velocity under hyperemic conditions, while baseline stenosis resistance (BSR) is calculated the same way but under resting conditions instead. Coronary flow reserve (CFR) is defined as the ratio between hyperemic and resting flow velocity.

 Microvascular resistance ( * Figure  5* , middle panel) measured at rest showed highly significant trends to fall with increasing stenosis severity, during both whole cycle and wave-free period specifically ( *P*_trend_ < 0.001). Hyperaemic MVR over the whole cycle was low for all stenosis severities, but showed a trend to increase in the most severe strata of stenosis severity ( *P*_trend_ = 0.01). This trend was not observed during the wave-free period under hyperaemic conditions, which remained consistent across all strata of stenosis severity ( *P*_trend_ = 0.89 for FFR and *P*_trend_ = 0.82 for anatomical stratification). The trend toward higher values of hyperaemic MVR over the whole cycle but not during the wave-free period, arises from an increasing MVR under the systole specifically ( Supplementary material online, Figure S2 ). Findings shown in * Figure  5* are maintained when post-PCI measurements are excluded or when only the post-PCI measurements are analysed ( Supplementary material online, Figure S3 ). 

**Figure 6:**
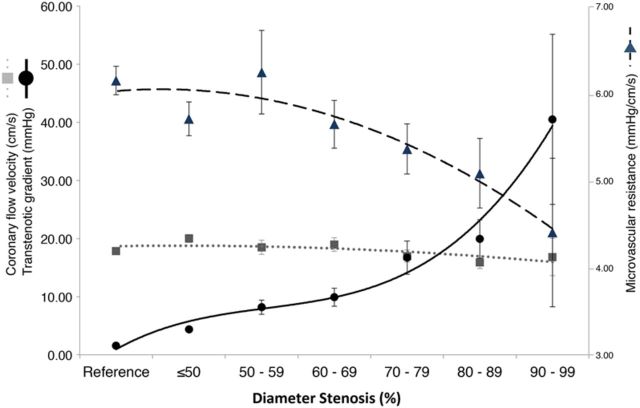
Mean TG, flow velocity and MVR data under resting conditions over the whole cardiac cycle, stratified according to angiographic stenosis severity. With progressive stenosis severity, TG increases, while flow velocity is maintained at a stable level by progressive compensatory reduction of MVR. Curves are fitted by 2nd, 3rd order and fractional polynomials.

 In contrast, TG ( * Figure  5* , lower panel) whether measured at rest or hyperaemia, had strong and significant relationships with stenosis severity and followed identical trends, also for the wave-free period ( *P*_trend_ < 0.001 for all assessments). Hyperaemic TG was strongly related to FFR ( *R*^2^ = 0.95, *P* < 0.001). The behaviour of flow, MVR and TG over the whole cycle under resting conditions for the entire study cohort is summarized in * Figure  6* . 

 A natural incremental hierarchy exists between the physiological states assessed: resting whole cycle, resting wave-free period, hyperaemic whole cycle and hyperaemic wave-free period physiology. This was true for flow velocity and TG ( *P* < 0.001 for both), while for MVR the same hierarchy exists in reverse ( *P* < 0.001). When stenoses had diameter stenosis >90% or FFR ≤ 0.50, the hierarchy was no longer valid with resting flow velocity exceeding hyperaemic and MVR being lower at rest than during hyperaemia. 

### Anatomical stenosis severity

 When analysed according to area stenosis or minimal lumen diameter the same physiological outcomes for flow velocity, MVR and TG were observed ( Supplementary material online, Table S1 ) as when analysed according to FFR or DS%. When data were analysed according to lesion length, resting flow velocity decreased numerically slightly but statistically significantly with increasing stenosis length ( Supplementary material online, Figure S4 ). Very long lesion lengths were the main contributor to this trend. In stenoses up to 40 mm long, resting wave-free flow velocity was preserved at 24.1 ± 12.6 cm/s; in stenoses over 40 mm, flow velocity was 19.1 ± 7.9, creating a significant trend ( *P* = 0.01) although not a significant difference in mean values by ANOVA ( *P* = 0.62) or *T* -test ( *P* = 0.15). The same was true for whole cycle resting flow velocity (18.3 ± 8.7 vs. 15.4 ± 1.9 cm/s, *P* = 0.23). Hyperaemic flow velocity similarly diminished from 39.4 ± 22.4 to 26.7 ± 18.8 cm/s, demonstrating a strong trend. Microvascular resistance appeared to be unrelated to lesion length, with no significant trends noted for rest or hyperaemia. Trans-stenotic pressure gradient was strongly related to length for all four physiological states ( *P*_trend_ < 0.001 for all). Minimal lumen diameter and area were in keeping with results as stratified according to FFR or DS% ( Supplementary material online, Table S2 ). 

 Overall trends and relationships observed remain unchanged when data are stratified according to the presence of diffuse and focal disease ( Supplementary material online, Figure S5 ) or according to singular and serial stenoses ( Supplementary material online, Figure S6 ). 

### Adenosine administration route

 Stratification of data by adenosine administration route according to FFR showed findings in keeping with the overall dataset for flow velocity and TG ( *P*_trend_ < 0.001 for all phases) ( Supplementary material online, Figure S7 ). Hyperaemic whole cycle MVR significantly increased with progressive stenosis severity for intracoronary administration ( *P*_trend_ = 0.04), but remained consistent for intravenous administration ( *P*_trend_ = 0.35). During the wave-free period, hyperaemic MVR was consistent for the intracoronary route ( *P*_trend_ = 0.17), but showed a trend to being lower with progressive stenosis severity with intravenous adenosine ( *P*_trend_ = 0.03). 

## Discussion

In this study, we describe the relationship between coronary flow velocity, TG, and MVR, estimated from measurements obtained over the whole cardiac cycle or selectively within the wave-free period, under resting and hyperaemic conditions.

 Firstly, we show that non-hyperaemic flow velocity remains constant across the full spectrum of stenosis severities. Secondly, this preservation of flow velocity is mediated by a reduction in resting MVR in response to increasing stenosis resistance. Thirdly, the maintenance of resting flow velocity occurs at the expense of distal coronary pressure, which falls with widening TG as stenosis severity increases. The capacity for resting gradients to increase while preserving flow velocity lends support to clinical use of invasive resting coronary pressure assessment to determine functional stenosis significance. Finally, we provide reference values of parameters used in physiological assessment of the coronary circulation stratified according to stenosis severity ( * Tables  2 and 3* ). 

### Auto-regulation ensures that resting blood flow remains stable

 Maintenance of resting coronary flow is regulated by endogenous adenosine release, changes in intrinsic myogenic tone, endothelial cell signalling and neurohumoral control, which combine to produce continuous auto-regulatory adaption of arteriolar vessel diameter. ^15^ In this study, we use invasively measured resting flow velocity and found that this was stable in human coronary arteries across a wide range of stenosis severities. While the resistance imposed by a stenosis rises according to the Hagen-Poiseuille equation, ^16^ we observe a reduction in MVR to compensate ( * Figure  6* summarizes the results for whole cycle resting conditions). This reduction is closely related to stenosis severity keeping resting flow stable and therefore, for the majority of moderate stenoses, a vasodilator reserve should be expected. This is observed by a reduction in MVR in the presence of a hyperaemic agent, such as adenosine. For stenoses with very little physiological impact, a large vasodilatory reserve is present meaning a large potential increase in flow during hyperaemia. In more significant stenoses, however, vasodilatory reserve will become progressively exhausted, with limited increase in flow response to an exogenous vasodilator. When a critical stenosis severity is reached (likely to be exceeding 85–90% diameter by formal QCA measurement, or FFR values <0.60), coronary auto-regulation becomes saturated with limited vasodilatory response to exogenous agents. When a stenosis is beyond this critical point, resting flow velocity is expected to fall. Clinically, this may manifest as angina on increasingly lower levels of exertion. 

### Anatomical and physiological markers of stenosis severity

 Cursory assessment of * Figure  4* may suggest that FFR and anatomical classification of stenoses are equivalent as the same patterns of change in flow and MVR are observed. However, as shown by many authors, there is a limited relationship between FFR and anatomical severity, which is confirmed in this cohort ( * Figure  2* ). The assessment is presented, not to state that anatomy and physiology are equivalent, rather because the overall trends are so strong that they are preserved even when the random scatter of the FFR-DS% relationship limits the potential relationship. Since anatomical assessment of stenoses remains mainstay and is readily understood by clinicians, it is appropriate to consider the underlying physiological response to anatomical parameters, despite the crude limitations of diameter stenosis. When study outcomes are analysed according to other parameters that describe lesion tightness (minimal lumen diameter, minimal lumen area, and area stenosis) similar findings are noted. This is also true for the presence of diffuse compared with focal disease and singular compared with serial stenoses. Resting flow velocity showed a trend to falling with very long lesion lengths (over 40 mm) but only showed a small change, while hyperaemic flow diminished significantly. * Figures  4* and *5* visually show that when stratified according to DS%, a remarkable overlap with respect to the TG for the 50–59 and 60–69% groups as well as the 70–79 and 80–89% groups is present. This observation reinforces that in stenosis of intermediate severity, physiological assessment is required to inform on haemodynamic significance. 

### The use of resting parameters to assess stenoses

 The stability of resting flow velocity for the majority of stenoses means that resting flow alone cannot distinguish between stenosis severities. However, since distal coronary pressure falls with increasing stenosis severity, a combined pressure and flow velocity measurement such as baseline stenosis resistance or a resting pressure-only index such as iFR, can distinguish stenosis severity. This implies that non-invasive imaging modalities such as positron emission tomography, that measure myocardial perfusion without knowledge of distal coronary pressure, require induction of the hyperaemic state to yield satisfactory diagnostic accuracy. ^17^

 The change in resting TG is predominantly driven by a change in MVR and resistance imposed by the stenosis. Since the impact of physiological vasodilation at rest on the proximal driving pressure is negligible, changes in distal pressure represent the true physiological impact of the stenosis on the distal coronary bed. Small gradients at rest suggest little compensatory vasodilatation is required, while large gradients indicate substantial compensation. For a stenosis to have a physiological impact upon the vessel, it should therefore have a gradient that is detectible at rest and the induction of hyperaemia will only exacerbate this gradient. Stenoses without a resting gradient which manifests only upon vasodilator administration more likely represents a situation in which the microcirculatory bed retains the capacity to dilate significantly and high flow velocities can be generated across a trivial stenosis with subsequent turbulence and pressure loss by the Bernoulli phenomenon. ^18^ These changes may manifest as a high coronary flow reserve (CFR), but a low FFR value. Human data with 10-year follow-up confirm that when such patients are deferred from PCI, the clinical event rate remains low, demonstrating a clear paradox between hyperaemic measurements of pressure and flow. ^19^

### Microvascular remodelling

 Resting MVR reduces with increasing stenosis severity. Theoretically, microcirculatory angiogenesis and arteriogenesis could explain this. ^20^ However, if this phenomenon applies, it would not be restricted to the resting situation and a substantial reduction in MVR would remain during hyperaemia in severe stenoses. Our results indicate that this is not the case and instead we confirm observations from smaller studies, that hyperaemic MVR increases in critical stenoses. ^8 , 10 , 21^ The increased hyperaemic MVR in severe stenoses is primarily a systolic phenomenon ( Supplementary material online, Figure S2 ). We presume this observed rise in hyperaemic MVR, can be attributed to the contribution of collateral circulation. Because collateral arteries connect with the receiving vessel distal to the position of the pressure–flow wire, flow supplied by the collateral arteries will not be detected, while collateral pressure is transmitted through the vessels and can be detected by the wire. Microvascular resistance is calculated as the ratio of distal pressure (elevated by collateral supply) and flow, and the calculated MVR will falsely rise accordingly. ^22^ Naturally, when using whole cycle pressure values, the contribution of the elevated pressures is higher than when using the lower diastolic pressure values as collateral pressure is elevated mainly during systole and much less so during diastole. ^23^ Moreover, in the intracoronary adenosine subgroup, a trend was observed towards higher MVR during hyperaemia whole cycle, while in the intravenous adenosine subgroup, hyperaemic whole cycle MVR was consistent across stenosis severities. In the intravenous subgroup, collateral supply may be diminished due to the coronary steal phenomenon during hyperaemia and thereby the MVR in these severe stenoses remains at normal values. However, this analysis has the limitation that coronary steal phenomenon might still apply in the intracoronary subgroup for the left coronary artery. Further work to assess the collateral flow or pressure during diastole is required to understand this in detail. 

### Clinical implications

 In this study, we provide flow velocity and resistance data from a wide spectrum of coronary stenoses and reference vessels. These data are valuable for accurate development and improvement of computer flow dynamics models. For current flow models, such as CT-FFR, data were derived from animals and small human studies without significant disease. ^24 , 25^ Our data demonstrate that unobstructed vessels have a mean CFR of 2.64 ± 0.76 in contrast to older data informing CT-FFR, which assumes flow rises of 3.5-fold. ^30^ Similarly, early CT systems assume resistance falls by 4.5-fold with adenosine, while our data show whole cycle MVR is reduced by 2.8-fold. 

Secondly, the data presented here provide reference values stratified according to stenosis severity for the most commonly used physiological indices. Exploration of less commonly used physiological parameters such as the instantaneous hyperaemic diastolic velocity–pressure slope (IHDVPS) and zero-flow pressure (ZFP) may be of future interest to better indicate their clinical applicability.

 Finally, the data support the concept that stenosis interrogation under resting conditions, as suggested by iFR, BSR, or baseline P _d_ /P _a_^10 , 12 , 13^ has clinical utility beyond comparisons of classification match with hyperaemic measures. Furthermore, our findings demonstrate that the wave-free period consistently provides a higher flow velocity and a lower MVR than assessment over the whole cardiac cycle at rest. This means that wave-free period gradients are consistently larger than over the whole cycle and iFR may provide greater sensitivity in moderate stenoses when compared with baseline P _d_ /P _a_ . To provide a definitive answer to which physiological index is preferable, randomized clinical outcome data are needed. 

## Conclusion

This large multicentre study of coronary pressure–velocity measurements shows that with progressive stenosis severity, TG rises, while resting coronary flow is maintained by compensatory reduction of MVR. This suggests that resting pressure indices can be used to detect the haemodynamic significance of coronary artery stenoses. Our results confirm the applicability of the general principles of coronary physiology determined in animals to patients with atherosclerotic lesions. The main difference observed is a relatively blunted response to hyperaemia as flow velocity rose to half what has been observed in animal models in vessels with <50% diameter stenosis.

## Limitations

 This study has a number of limitations. Volumetric flow was not assessed because of the limitations of accurate stenosis and vessel dimension calculation, as well as determining the mass of the subtended myocardium which can only be estimated from angiographic parameters. Since vessels taper, flow velocity will fall less than volumetric flow and without knowledge of subtended mass, flow velocity might be preferable to volumetric flow. ^26–28^

Wedge pressure was not routinely measured and therefore definitive assessment of the impact of collaterals on the results cannot be made. However, measurements were not made in vessels with visible collaterals.

 While reference vessels were free of angiographic disease, intravascular ultrasound studies demonstrated significant burden of atherosclerosis in apparently unobstructed coronary arteries. ^29^ Diffuse atherosclerosis can cause pressure loss and this may account for the wide-range of FFR values observed in reference vessels (lowest obtained 0.85). It remains uncommon to routinely perform intravascular imaging in unobstructed vessels and therefore, together with the large number of unobstructed vessels, our findings should be applicable to patients undergoing coronary angiography. 

 Although we stratified data according to FFR and QCA DS%, both are imperfect measures. In the presence of microcirculatory dysfunction, FFR may underestimate true haemodynamic stenosis significance. ^30^ Quantitative angiography provides limited information of the physiological impact of a given stenosis. However, both measures are easy to comprehend and familiar to clinicians providing a familiar conceptual framework to interpret the data. 

 Finally, it must be borne in mind, however, that our results are inferred on group basis and heterogeneous factors such as microvascular dysfunction and diffuse epicardial disease could obscure these findings on a patient-specific level. ^31 , 32^ Theoretically, however, any factor that impairs auto-regulatory responses to a stenosis could also impact upon microcirculatory responses to vasodilators such as adenosine. When there are discrepancies between resting and hyperaemic factors, it remains unclear which parameters provide prognostic information. Randomized clinical outcome studies are currently being undertaken to assess the safety and performance of resting parameters to guide revascularization. ^33 , 34^

## Supplementary material

 Supplementary material is available at European Heart Journal online. 

## Authors’ contributions

S.N. performed statistical analysis; J.D., N.v.R. handled funding and supervision. S.N., G.d.W., S.S., T.v.d.H., R.P., M.E.-P., M.v.L., M.M., I.D., P.K., J.E., J.P., J.D., and N.v.R. acquired the data. S.N., G.d.W., J.D., and N.v.R. conceived and designed the research. S.N., G.d.W., J.D., and N.v.R. drafted the manuscript. S.S., T.v.d.H., R.P., M.E.-P., M.v.L., M.M., I.D., P.K., J.E., and J.P. made critical revision of the manuscript for key intellectual content.

## Funding

This work was supported by the Medical Research Council (UK), British Heart Foundation and the National Institute for Health Research Imperial Biomedical Research Centre (to S.N., S.S., and R.P.) and the Institute for Cardiovascular Research of the VU University of Amsterdam (ICaR-VU) (to G.d.W. and N.v.R.). Funding to pay the Open Access publication charges for this article was provided by the VU University Medical Center.

**Conflict of interest:** J.E.D. and J.J.P. report consultancy work for Volcano Corporation. J.E. reports consultancy work for Volcano Corporation and St Jude Medical. J.E.D. holds intellectual property which is under license. 
